# Large‐scale molecular diet analysis in a generalist marine mammal reveals male preference for prey of conservation concern

**DOI:** 10.1002/ece3.4474

**Published:** 2018-09-15

**Authors:** Dietmar Schwarz, Sara M. Spitzer, Austen C. Thomas, Christa M. Kohnert, Theresa R. Keates, Alejandro Acevedo‐Gutiérrez

**Affiliations:** ^1^ Department of Biology Western Washington University Bellingham Washington; ^2^ Department of Zoology and Marine Mammal Research Unit Institute for the Oceans and Fisheries University of British Columbia Vancouver British Columbia Canada; ^3^ Smith‐Root Vancouver Washington; ^4^ Present address: Illumina Inc. San Diego California; ^5^ Present address: Department of Ocean Sciences University of California Santa Cruz California

**Keywords:** diet analysis, DNA metabarcoding, marine mammals, predator prey interactions, sex identification

## Abstract

Sex‐specific diet information is important in the determination of predator impacts on prey populations. Unfortunately, the diet of males and females can be difficult to describe, particularly when they are marine predators. We combined two molecular techniques to describe haul‐out use and prey preferences of male and female harbor seals (*Phoca vitulina*) from Comox and Cowichan Bay (Canada) during 2012–2013. DNA metabarcoding quantified the diet proportions comprised of prey species in harbor seal scat, and qPCR determined the sex of the individual that deposited each scat. Using 287 female and 260 male samples, we compared the monthly sex ratio with GLMs and analyzed prey consumption relative to sex, season, site, and year with PERMANOVA. The sex ratio between monthly samples differed widely in both years (range = 12%–79% males) and showed different patterns at each haul‐out site. Male and female diet differed across both years and sites: Females consumed a high proportion of demersal fish species while males consumed more salmonid species. Diet composition was related to both sex and season (PERMANOVA:* R* ^2^ = 27%, *p *<* *0.001; *R* ^2^ = 24%, *p* < 0.001, respectively) and their interaction (PERMANOVA:* R* ^2^ = 11%, *p *<* *0.001). Diet differences between males and females were consistent across site and year, suggesting fundamental foraging differences, including that males may have a larger impact on salmonids than females. Our novel combination of techniques allowed for both prey taxonomic and spatiotemporal resolution unprecedented in marine predators.

## 1 INTRODUCTION

Predators can have important effects on prey populations (Holt, 2008; Marshall, Stier, Samhouri, Kelly, & Ward, 2016). One important factor in evaluating a predator's effect on its prey is the degree of predator specialization, and the effects of predators on prey vary depending on where a particular predator falls along the specialist–generalist continuum (Jiang & Morin, 2005). However, a predator population with a generalist diet spectrum at the population level may in fact be composed of a mixture of individual specialists (Bolnick et al., 2003) and such within population variation may have important ecological effects (Bolnick et al., 2011). One of the most commonly described forms of specialization within a population is sexual segregation in foraging (Ruckstuhl, 2007; Wearmouth & Sims, 2008). Sexes may differ in the width of their diet spectra and therefore in the degree of specialization. In addition, the overlap between the diet spectra of the sexes may take different forms. Males and females may be distinct specialists and show little overlap in the prey they utilize. This may be particularly common in species with strong sexual dimorphism and habitat segregation (e.g., sea lions, Le Boeuf et al., 2000) The diet spectrum of one sex could be also be completely nested within the spectrum of the other, meaning that one sex would be relatively more generalist than the other. This can be observed in cases where females have additional nutritional needs due to reproduction (e.g., both male and female adult mosquitoes consume nectars, but only females are blood feeders, Gu, Müller, Schlein, Novak, & Beier, 2011). Differential nutritional needs during or behavioral constraints associated with the rearing of offspring may further result in seasonal variation in sexual segregation in foraging (e.g., seabirds, Phillips, McGill, Dawson, & Bearhop, 2011). Ignoring sexual segregation in foraging may have important consequences on understanding the effect of predators on their prey. And in cases where the prey is of conservation or economic concern it may even result in applied consequences when management decisions are made based on naïve assumptions about intrapopulational differences in diet (Bolnick et al., 2003, 2011). Ignoring intrapopulational variation in predation may, for example, result in overestimating the mean effect of a predator population on its prey (Okuyama, 2008).

Unfortunately, information about sex‐specific foraging preferences can be notoriously difficult to obtain. This is particularly true for predators that are not clearly sexually dimorphic and whose foraging behavior is difficult to observe due to a secretive or aquatic lifestyle (Wearmouth & Sims, 2008). Moreover, in studying a predator that has a generalist and opportunistic foraging behavior, it is important to describe its diet with a high degree of taxonomic and spatiotemporal resolution (Thomas, Nelson, Lance, Deagle, & Trites, 2017). Marine mammals exemplify the difficulties in understanding sex‐specific differences in foraging behavior of predators. Their aquatic lifestyle makes direct observation difficult and their protected status—in addition to logistical and financial constraints—limit the sample size of invasive methods. At the same time, marine mammals as a population may prey on a great diversity of species and tend to respond to spatiotemporal pulses in prey availability and/or profitability (Lance, Chang, Jeffries, Pearson, & Acevedo‐Gutiérrez, 2012; Thomas, Lance, Jeffries, Miner, & Acevedo‐Gutiérrez, 2011). Hence, they may be viewed as hypergeneralists.

Several approaches have been used to estimate sex‐specific diet differences in marine mammals (Bowen & Iverson, 2013). Most often, foraging sexual segregation has been inferred from differences in movement and diving patterns between females and males (Wearmouth & Sims, 2008). While these approaches can provide valuable information about differences in foraging behavior, the taxonomic identities and relative quantities of prey consumed remain unknown. Further, sex‐specific differences in movement patterns may also reflect other ecological or physiological reasons (e.g., Harvey, Côté, & Hammill, 2008; Le Boeuf et al., 2000). Stable isotope ratios (Bowen & Iverson, 2013; Kelly, 2000; Phillips & Gregg, 2003) and fatty acid analyses (Bowen & Iverson, 2013; Bromaghin, 2017; Budge, Iverson, & Koopman, 2006) have also been used to infer sex‐specific differences in diet, but the prey taxonomic resolution of both techniques is limited. Most importantly, both stable isotope analysis and fatty acid analyses are highly invasive and/or difficult as they require tissue samples and often result in small sample sizes that may not be reflective of the entire population. Recovering hard parts of prey remains by examining stomach contents results in an increased taxonomic resolution of prey consumed (Bowen & Iverson, 2013). However, at present, the analysis of stomach contents is largely limited to dead individuals that wash up on shore, resulting in small sample sizes. In contrast, diet analysis from fecal samples is a relatively noninvasive method that allows for large sample sizes to be collected (Bowen & Iverson, 2013). Unfortunately, unlike the invasive methods above that involve the capture of animals, traditional fecal analysis does not allow for partitioning diet by sex. On the other hand, scat does contain DNA left by the depositor, which can be used to sex the depositor by targeting sex‐linked markers like SRY or ZFX/ZFY (Matejusová et al., 2013; Reed, Tollit, Thompson, & Amos, 1997). When paired with conventional diet analysis from scat via hard parts, genetic sex determination assays can provide sex‐specific diet information in marine mammals (Wilson, 2015). While providing a greater taxonomic resolution of prey items than stable isotope or fatty acid analyses, it is nearly impossible to morphologically identify hard parts to the species level in groups of closely related prey species, such as Pacific salmon (*Oncorhynchus* spp) or rockfish (*Sebastes* spp) (Harvey, 1989; Phillips & Harvey, 2009; Tollit, Heaslip, Barrick, & Trites, 2007). In addition, some types of harbor seal foraging, such as “belly biting” of salmon may leave no hard parts in the scat as only soft tissues are consumed (Hauser, Allen, Rich, & Quinn, 2008). However, molecular bar coding of prey does allow for the species‐level resolution of prey items (Bowen & Iverson, 2013; Bowles & Trites, 2013; King, Read, Traugott, & Symondson, 2008). The combination of molecular bar coding with hard parts analysis can even provide information about which age or life stage of a particular prey species was consumed (Thomas et al., 2017). This information is crucial when predation on different prey life history stages has different impacts on the prey population as is, for example, the case with Pacific salmon species (Chasco et al., 2017a,2017b; Thomas et al., 2017).

Questions regarding the foraging ecology of marine mammals are best examined by a combination of different techniques (e.g., Jeanniard‐du‐Dot, Thomas, Cherel, Trites, & Guinet, 2017). Here, we present a novel combination of noninvasive techniques that use scat DNA for both sex determination of the predator and high taxonomic resolution molecular bar coding of the prey.

We apply this methodology to harbor seals (*Phoca vitulina*) in the Strait of Georgia, Canada, that are an excellent study system to describe the diet of males and females using molecular techniques and determine the importance of sexual segregation in foraging. Harbor seals are an abundant and common species in the Salish Sea, the inland waters of the Pacific Northwest (Jeffries, Huber, Calambokidis, & Laake, 2003; Olesiuk, 2009). Here, they consume both out‐migrating juvenile salmon and returning salmon adults (Thomas et al., 2017) and this predation is of special economic and conservation concern (Marshall et al., 2016). In contrast to the historical extirpations and declining trends for culturally, commercially, and recreationally significant salmon runs in the region (Ford, 2011; Gustafson et al., 2007), harbor seals have recovered since the early 1970s (Jeffries et al., 2003; Olesiuk, 2009) and increased salmon consumption (Chasco et al., 2017a,2017b). Fisheries scientists and managers are therefore interested in quantifying the impact that harbor seal predation has on salmon populations. Consistent differences in salmon consumption between seals of different sex could have important consequences for understanding these impacts. The two sexes differ in their energy needs (Howard, Lance, Jeffries, & Acevedo‐Gutiérrez, 2013) and thus cannot be regarded as equivalent in bioenergetic models if they consume prey in different proportions; ignoring these differences may result in errors in consumption rate estimates. In addition, the sex ratio may vary in space and time as sexes respond differently to prey availability or have different reproductive constrains (e.g., Kovacs, Jonas, & Welke, 1990; Thompson, 1989), thus further introducing errors into consumption models. Male and female seals may also occupy different positions in marine food webs involving salmon and have different direct or indirect effects on salmon, thereby resulting in potentially unexpected complications for ecosystem models that seek to understand the impact of salmon predation (Bjorkland et al., 2015).

In pinnipeds (the clade of marine mammals consisting of seals, sea lions, and the walrus) sex‐specific differences in foraging behavior have been reported in multiple species (Wearmouth & Sims, 2008). Sex‐specific differences in movement and diving patterns both during and outside the breeding season are common, with males tending to move farther and spend more time foraging than females (Wearmouth & Sims, 2008). The best documented cases of sex‐specific differences in foraging and diet come from species with pronounced sexual size dimorphism such as gray seals (*Halichoerus grypus*) and northern elephant seals (*Mirounga angustirostris*) (Beck, Iverson, Bowen, & Blanchard, 2007; Breed, Bowen, McMillan, & Leonard, 2006; Le Boeuf et al., 2000). For instance, in the Baltic Sea, a preference for raiding salmon traps has been documented in male gray seals as a result of their larger size (Königson, Fjälling, Berglind, & Lunneryd, 2013). In addition to the energetic demands of size itself, differences in behavioral trade‐offs that are linked to reproduction are likely the causes of differences in foraging (e.g., Breed et al., 2006). Harbor seals do not show such pronounced sexual dimorphism; in a population believed to have the largest differences between the sexes, males are on average 9% longer and 25% heavier than females (Lydersen & Kovacs, 2005). Nevertheless, this size difference significantly impacts foraging behavior, prey consumption, and energetic models (Bjorkland et al., 2015; Howard et al., 2013; Thompson, Mackay, Tollit, Enderby, & Hammond, 1998).

Life history and ecological constraints also seem to influence sex‐specific foraging in harbor seals. Due to the unique reproductive costs between the sexes, harbor seal mothers continue to forage while pupping (Boness, Bowen, & Oftedal, 1994) and as pups often accompany their mothers on these trips, foraging time tends to be shorter and restricted to feeding areas close to the haul‐out site (Bowen, Bonness, & Iverson, 1999; Newby, 1973). Because male harbor seals do not participate in parental care, they are free to travel widely, presumably to more ideal foraging locations (Van Parijs, Thompson, Tollit, & Mackay, 1997), potentially leading to sex‐specific differences in prey consumption. However, males also restrict their foraging range near the end of the lactation period, which may result in a decrease in diet diversity for males during this time as well (Coltman, Bowen, Boness, & Iverson, 1997; Van Parijs et al., 1997). Indeed, a previous study characterizing stable isotopes in a small sample of harbor seals in the Salish Sea suggested that males tend to consume salmon whereas females consume a variety of benthic species (Bjorkland et al., 2015).

In this study, we performed DNA‐bar coding analysis and qPCR on harbor seal fecal samples to determine the identity, estimate prey species proportions in diet, and the sex of the harbor seal, respectively. Other pinniped studies have used molecular techniques to determine the diet of one sex (Jeanniard‐du‐Dot et al., 2017; Peters et al., 2015) or the diet of the species without differentiating the sex (Hui, Morita, Kobayashi, Mitani, & Miyashita, 2017; Kvitrud, Riemer, Brown, Bellinger, & Banks, 2005; Parsons, Piertney, Middlemas, Hammond, & Armstrong, 2005; Thomas et al., 2017; Wright, Riemer, Brown, Ougzin, & Bucklin, 2007). To our knowledge, this is the first study in pinnipeds that incorporates these two molecular methods to differentiate males and females and estimate their diet from scat. Using these relatively noninvasive molecular methods, we obtained a consistently large sample size over long periods of time, which allowed us to generate more accurate results than other popular diet methods, and unambiguously describe sex‐specific harbor seal diet.

## 2 MATERIALS AND METHODS

### 2.1 Scat collection

Harbor seal scat collections for this study and molecular diet analyses are described in detail in Thomas et al. (2017). Briefly, harbor seal scat samples were collected from two estuarine haul‐out sites, Comox and Cowichan Bay, in the Strait of Georgia, British Columbia, Canada. The estimated haul‐out population sizes were 121 at Comox and 167 at Cowichan bay based on a survey conducted in August 2008 (Olesiuk, 2009). Scat collection was performed at each site in 2012 and 2013 during the harbor seal prepupping, pupping, breeding and molting seasons (April–November). The collection period was also timed to correspond with juvenile salmon out‐migrations (spring) and adult salmon spawning (fall). Most pink salmon in the study region belong to lineages that return in odd numbered years resulting in characteristic “pink years” (Krkosek, Hilborn, Peterman, & Quinn, 2010). Strong returns are followed by low returns of pink salmon during even numbered years and our study captured one such cycle with 2013 being a pink year. Scat samples were either preserved immediately in 95% ethanol or stored in a −20°C freezer <6 hr from collection. Samples were thawed, manually homogenized, and hard parts (e.g., bones) were removed prior to DNA extraction from the scat matrix material. Extracted scat gDNA samples were stored at −80°C until needed for DNA metabarcoding and qPCR analysis, at which time they were stored at −20°C. The harbor seal scats were collected under Fisheries and Oceans Canada Marine Mammal Research License (MML 2011‐10) and a University of British Columbia Animal Care Permit (A11‐0072).

### 2.2 Diet analysis via metabarcoding and hard part analysis

DNA metabarcoding analysis was performed as described in Thomas et al. (2017) to quantify the diet proportions of each fish species. Briefly, the small subunit ribosomal RNA sequence was used as the metabarcoding marker (~260 bp) and the PCR primers were designed to capture both fish and cephalopod prey species. Scat sample amplicons were prepared for sequencing using the Illumina TruSeqTM DNA sample prep kit and sequenced on an Illumina MiSeq sequencer. Prey species were identified by nucleotide BLAST using a custom reference library of fish and cephalopod DNA sequences. We were also able to specify whether salmon DNA came from an adult or juvenile by combining DNA and hard parts data (Thomas et al., 2017). The sizes of prey bones were used to estimate the life stage of salmon consumed, while DNA metabarcoding was used to determine specific proportions of each salmon species in the diet (see Thomas et al., 2017 for details).

### 2.3 Seal sex determination via qPCR

Quantitative polymerase chain reaction (qPCR) was used to determine the sex of the individual that deposited each scat using a modified version of the seal‐specific assay developed by Matejusová et al. (2013). The modified version is described in detail by Rothstein (2015). Briefly, we performed two Taqman qPCR reactions that targeted the paralogous zinc finger x (ZFX) and zinc finger y (ZFY) genes, respectively, to determine seal sex. ZFX acted as a positive control, as all scat samples should contain the ZFX gene, while the presence or absence of ZFY would determine the sex. ZFX and ZFY probes were custom‐synthesized by Applied Biosciences and were diluted to 10× concentration. We used 2× Taqman Gene Expression Master Mix from Applied Biosciences. ZFX and ZFY Master Mixes were made with 10 μl of 2× Taqman Gene Expression Master Mix for every 1 μl of 10× ZFX or ZFY probe. The optimized qPCR reaction was comprised of 11 μl ZFX or ZFY Master Mix with 9 μl of gDNA or PCR water. The thermocycler protocol was as follows: one holding cycle (50°C for 2 min, 95°C for 10 min) followed by 60 cycles of denaturation and annealing/extension (95°C for 15 s, 60°C for 1 min). We ran two ZFX and two ZFY replicates for each sample. Each qPCR reaction profile was manually inspected for the presence of an amplification curve. If none of the two ZFX replicates amplified in a particular sample, we considered sexing to have failed and the sample was excluded from further analysis (21% of initial samples). If one or two of the ZFY replicates showed amplification, the sample was classified as a male. If none of the ZFY replicates showed amplification, the sample was classified as a female. This procedure also excluded a small number of samples with ZFY but not ZFX amplification. Given the, albeit small, chance of false positive ZFY amplification (s. below), we erred on the side of caution and did not classify these samples as male. Each 96‐well reaction plate included a positive known male and positive known female control with two replicates each and four nontemplate controls with PCR‐grade water. Five scat samples from known males and five scat samples from known females that were collected from captive animals at the Vancouver Aquarium, Vancouver, British Columbia, Canada, and the Point Defiance Zoo and Aquarium, Tacoma, Washington, USA, respectively, served as positive controls. In an initial analysis, all ten samples from captive animals of known sex were positive for the ZFX marker and all male samples were positive in each of the two replicates for ZFY, whereas all female samples were negative for each of the two replicates for ZFY. In subsequent >100 replicate amplifications of these same samples, the false negative rate for ZFX was 5% and the false negative and false positive rates for ZFY were 4% and 2%, respectively. We decided against applying a maximum Ct value threshold because we did not attempt to quantify the template DNA but were instead scoring presence and absence of amplification for each marker. Our scoring method made it more likely for males to be classified as females than vice versa because two false negative replicates at ZFY resulted in misclassification of males as females, whereas two false negatives at ZFX led to the exclusion of the sample from analysis. Cases in which only one of two replicates for ZFY amplified can be used as a crude estimate for the false negative rate in ZFY if we assume that all these instances are a combination of a true positive and a false negative. Our false negative rate estimate then becomes ½ times the number of individuals classified as males with one of two ZFY amplifications divided by the total number of individuals classified as males. For our data, this estimate is 13.4%, resulting in a posterior probability that a sample is male if two of two replicates for ZFY fail of 1.8% at equal proportions of males and females in the population. When the proportion of males range from 0.1 to 0.9,the corresponding posterior probabilities range from 0.4% to 3.2%.

### 2.4 Statistical analyses

To analyze seal sex ratio at the haul‐out sites, we calculated the proportion of males for each monthly sample and compared generalized linear models (binomial error with logit transformation) in software R (R Core Team, 2016) to identify the combination of factors that best accounted for the observed variation in the proportion of males between samples. Month, site, and year were all potential predictors sex ratio differences between samples. Plotting the sex ratios further suggested different intra‐annual trends among the two sites, and we therefore also examined the effect of a month * site interaction term as well as the performance of the full model including all possible interactions between the three factors (Table 2). We checked for overdispersion by taking the ratio of residual deviance and degrees of freedom and used delta AICc and the resulting probability of each model (*w*_*i*_) as our main criteria for model selection (Burnham, Anderson, & Huyvaert, 2011). We further calculated *R* ^2^ values for each model by 1‐(Residual Deviance/Null Deviance). We opted for the greater temporal resolution of monthly sex ratio estimates even though the number of month/site/year data points meant that many sex ratios were based on small to moderate sample sizes (Table 1).

**Table 1:** Diet of harbor seals in Comox and Cowichan Bay during 2012–2013. Values are percentage of prey DNA in scat See Data Accessibility to download file with the full list of prey taxa in the diet

Site	Year	Season	Sex	Sample size	Salmoniformes	Gadiformes	Clupeiformes	Perciformes	Scorpaeniformes	Pleuronectiformes	Gasterosteiformes	Other
Comox	2012	E	M	56	0.228	0.217	0.380	0.076	0.003	0.023	0.006	0.068
			F	28	0.097	0.184	0.327	0.125	0.124	0.085	0.014	0.044
		L	M	28	0.407	0.160	0.275	0.096	0.058	0.001	0.001	0.002
			F	42	0.112	0.069	0.296	0.240	0.207	0.048	0.000	0.028
	2013	E	M	37	0.193	0.426	0.258	0.030	0.027	0.000	0.009	0.057
			F	28	0.129	0.251	0.231	0.071	0.059	0.184	0.053	0.021
		L	M	26	0.626	0.105	0.174	0.073	0.012	0.003	0.003	0.003
			F	37	0.282	0.077	0.261	0.192	0.115	0.068	0.000	0.005
Cowichan	2012	E	M	27	0.069	0.307	0.455	0.037	0.041	0.000	0.003	0.087
			F	27	0.196	0.071	0.505	0.010	0.067	0.072	0.045	0.034
		L	M	40	0.438	0.257	0.194	0.040	0.044	0.005	0.000	0.022
			F	41	0.218	0.270	0.293	0.046	0.142	0.007	0.001	0.024
	2013	E	M	15	0.115	0.465	0.289	0.070	0.016	0.008	0.035	0.002
			F	38	0.146	0.192	0.444	0.027	0.048	0.030	0.074	0.039
		L	M	31	0.446	0.317	0.175	0.010	0.019	0.001	0.000	0.032
			F	46	0.162	0.258	0.306	0.176	0.055	0.019	0.015	0.009
All samples			M	260	0.315	0.282	0.275	0.054	0.028	0.005	0.007	0.034
			F	287	0.168	0.171	0.333	0.111	0.102	0.064	0.025	0.025

To analyze sex‐specific seal diet, for each scat sample we divided the sequence reads for each prey taxon by the total number of sequence reads to normalize for differences in sequencing coverage between samples. We pooled fish prey taxa by order for our first set of analyses, which consisted of permutational analysis of variance (PERMANOVA), principal component analysis (PCA), and calculation of Shannon's diversity indices. For PERMANOVA and PCA, we only chose orders with a mean diet proportion across the entire dataset of >0.01, leaving us with a group of seven common orders (Table 1). We tested for overdispersion of the Bray–Curtis distances among all individual samples across all sites, seasons, and years using the betadisper function in the R package vegan (Oksanen et al., 2016) and found that female individual samples were significantly overdispersed when compared to males (permutation test: *p *=* *0.002, 999 permutations), thereby violating an important assumption of PERMANOVA. Consequently, we pooled the samples as follows: we averaged the diet proportions from each prey order across all scats that were assigned to the same sex (male or female) and were collected during the same season (early = April–July or late = August–November) in the same year (2012 or 2013) and at the same site (Comox or Cowichan Bay) (Table 1). This resulted in 16 sample pools that we used for all analyses described in the following with exception of prey species level diet comparisons (s. below). The dispersion of male and female pools was not significantly different.

We tested for diet differences at the prey order level relative to sex, season, site and year, as well as for all two‐way interactions between the factors with a PERMANOVA (10,000 permutations) as implemented by the function adonis in the R package vegan (Oksanen et al., 2016). To visualize the patterns by which male and female diet differ, we conducted a centered and scaled PCA using the prcomp function in R (R Core Team, 2016).

We compared diet diversity at the prey order level using Shannon diversity indices that we calculated across the mean diet proportions of all 16 observed orders, including rare orders with a mean diet proportion of <0.01 (see Data Accessibility section), for each of our pooled samples using the diversity function in R package vegan (Oksanen et al., 2016). We examined the effects of sex, season, site, and year on Shannon diversity index with a generalized linear models (Gaussian error) in software R (R Core Team, 2016). Among the four single factor models, the model using only sex as an explanatory variable performed best. We then expanded our models to include all additive combinations of the other three factors and sex among which sex + season had the best support. Finally, we tested three models in which we added an interaction of sex with season, site, and year to the sex + season model. Models were evaluated using the criteria described above. We also compared the prey species richness for each sex/season/site/year combination by counting prey species with a minimum diet proportion of 0.01 in each sample pool and built generalized linear models using the same procedure (Supporting Information Appendix S2 and S3).

We used the R package DEseq2 (Love, Huber, & Anders, 2014) to test for sex‐specific differences in the diet proportions of each individual prey species separately for each of the four site/year combinations. (A separate analysis of the eight season/site/year combination produced very similar results.) We included all species with a mean diet proportion of >0.01 in at least one of the site/year combinations and fitted a negative binomial generalized linear model for each site/year using default settings. This fit calculated log2 fold changes with females as the reference group. We then tested the significance of the model coefficients with a Wald test. To prepare data for model building, reads for all prey items were transformed (*x* + 1) to eliminate instances of zero sequence reads that interfered with analysis in DEseq2. This transformation minimally impacted relative percentages of prey in samples with high read counts, thus samples with fewer than 60 total reads (*n *=* *51) were excluded from model building for this part of the analysis.

## 3 RESULTS

### 3.1 Harbor seal haul‐out use

**Figure 1:**
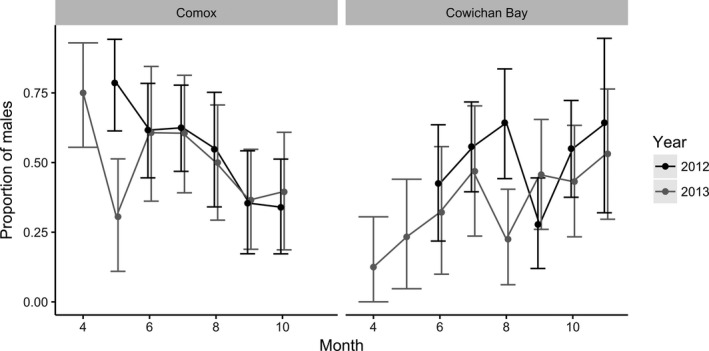
Proportion of male harbor seals in Comox and Cowichan Bay haul‐out sites in 2012 (black) and 2013 (gray). Error bars represent 95% binomial confidence intervals (Dorai‐Raj, 2014)

Sex determination succeeded in 287 scat deposited by harbor seal females and 260 deposited by males (Table 1, Supporting Information Appendix S1). The sex ratio between monthly samples at different sites and in different years differed widely (range = 12%–79% males) and showed different trends at Comox and Cowichan Bay that were largely consistent in both years (Figure 1). Whereas the early season had a lower proportion of males than the late season at Cowichan Bay, the pattern was reversed at Comox. Including the interaction between site and month greatly improved the GLM explaining variation in sex ratio, with the addition of year further improving the model (Table 2). The fluctuations in sex ratio were the result of changes in the counts of scats from both sexes as opposed to fluctuations in only one sex while the other sex maintained a constant sample size (Supporting Information Appendix S1).

**Table 2:** Comparison of general linear models of differences in sex ratio between samples

Model	AIC	AICc	Δ AICc	*w*_*i*_	*R* ^2^
Month + Site + Year + (Month*Site)^a^	129.4	132.3	0	0.713	0.505
Month + Site + (Month*Site)	132.6	134.4	2.1	0.254	0.423
Month + Site + Year + (Month*Site) + (Month*Year) + (Site*Year) + (Month*Site*Year)	130.5	138.5	6.2	0.033	0.585
Site + Year	147.6	148.6	16.3	<0.001	0.150
Month + Site + Year	148.0	149.8	17.5	<0.001	0.176
Month + Year	149.4	150.4	18.1	<0.001	0.121
Year	150.0	150.5	18.2	<0.001	0.079
Site	150.1	150.6	18.3	<0.001	0.077
Month + Site	151.0	152.1	19.8	<0.001	0.095
Month	152.9	153.4	21.1	<0.001	0.032

a Coefficients (95% coefficient confidence intervals, *p*‐values) for each variable in the best supported model “Month + Site + Year + (Month*Site)”: intercept: 1.868 (0.850–2.922, *p* < 0.001), Month: −0.273 (−0.411 to −0.140, *p* < 0.001), Site (Cowichan): −3.710 (−5.281 to −2.185, *p* < 0.001), Year (2012): 0.399 (0.053–0.748, *p* = 0.024), Month*Site(Cowichan): 0.437 (0.246–0.633), *p* < 0.001).

### 3.2 Harbor seal sex‐specific diet at the order level

**Figure 2:**
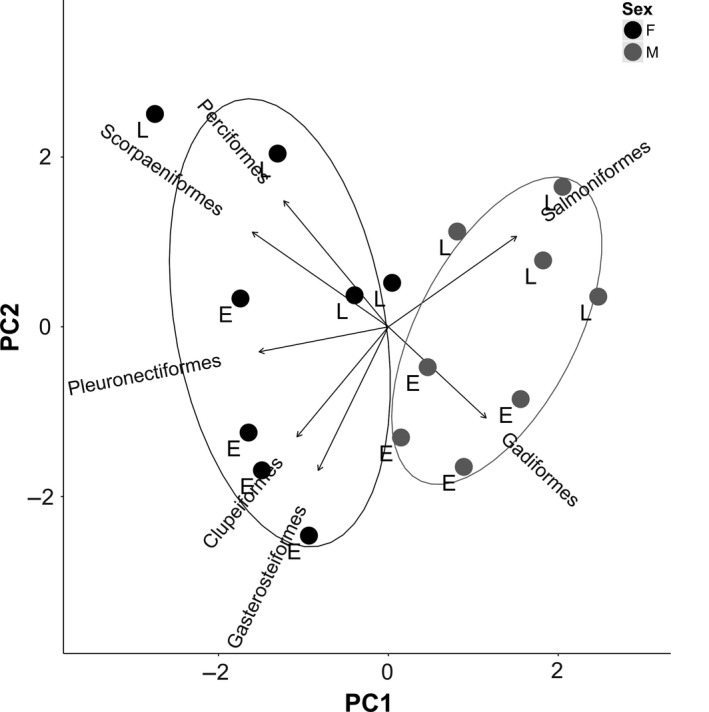
Principal component analysis of the harbor seal diet proportions of the most common prey fish orders. Each data point represents the mean diet proportions for seals of a given sex at one of the two study sites in one of the two study years during either early or late season. Females = black, Males = gray. Letters indicate early (E) or late (L) season. Arrows indicate the loadings for the different axes by prey fish order. Normal data ellipses representing 68% of the probability distribution around the mean are shown for males and females

**Figure 3:**
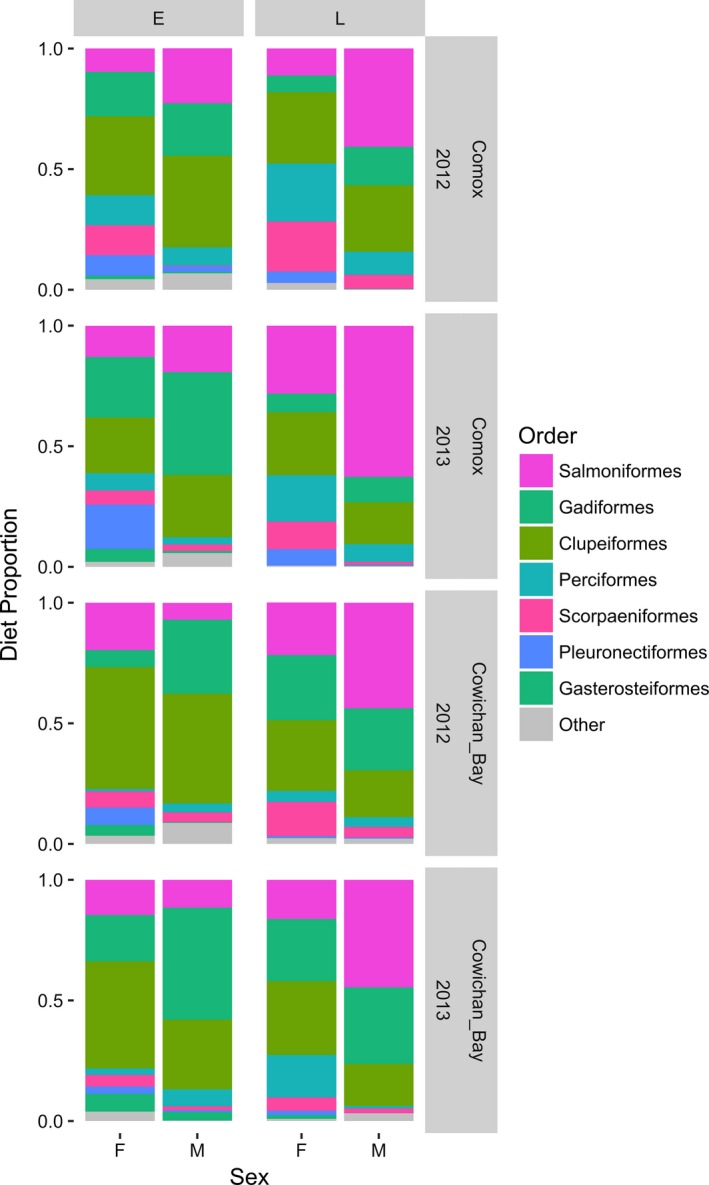
Harbor seal diet proportions of seven prey orders by Sex (M/F), Season (E/L), Site (Comox/Cowichan Bay), and Year (2012/2013). The diet proportions of prey orders with a mean annual diet proportion of >/= 0.01 in at least one site/year combination are shown separately. Other = rare prey orders with < 0.01 mean annual diet proportion in all site/year combinations

**Figure 4:**
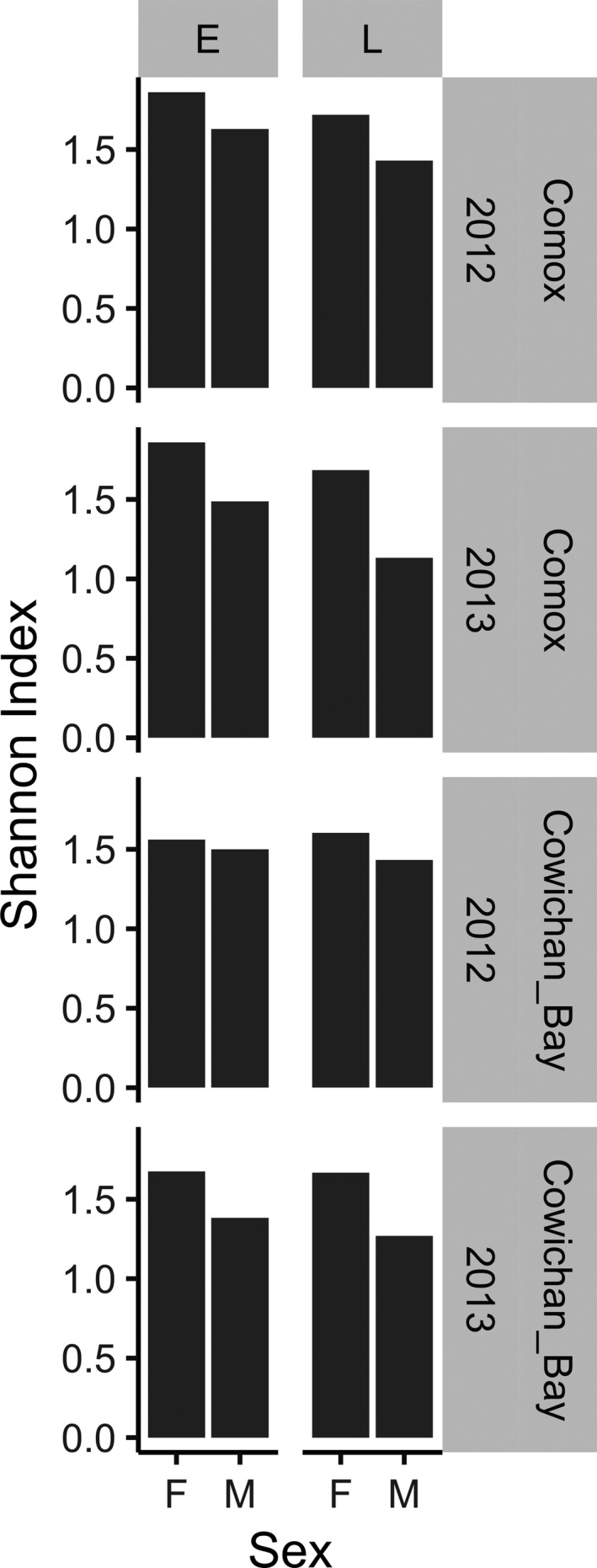
Harbor seal Shannon indexes of prey order diet diversity by sex (M/F), season (E/L), site (Comox/Cowichan Bay) and year (2012/2013)

Males and females showed strong and consistent differences in their diet across both years and sites (Figures 2 and 3). The differences were driven by females having a higher proportion of Scorpaeniformes, Perciformes, Pleuronectiformes, and Gasterosteiformes in their diet while males had greater diet proportions of Gadiformes in the early season and Salmoniformes in the late season (Figures 2 and 3). Differences between the sexes were more pronounced in the late season as the male diet contained less Pacific herring (*Clupea pallasii*) and more salmon, whereas females increased their use of Scorpaeniformes and Perciformes in the late season, particularly at Comox (Figure 3). Sex and season were the most important factors in explaining variation in diet among the 16 pooled samples (PERMANOVA: *R* ^2^ = 27%, *p *<* *0.001 and *R* ^2^ = 24%, *p *<* *0.001, respectively, Table 3). The interaction between sex and season was significant as well (PERMANOVA: *R* ^2^ = 11%, *p *<* *0.001, Table 3). There was also a marginally significant interaction between sex and site (PERMANOVA: *R* ^2^ = 3%, *p* = 0.096), driven by greater proportions of Scorpaeniformes, Perciformes, and Pleuronectiformes in female scats at Comox; however, there was no significant interaction between sex and year (Table 3). Although none of the taxa were exclusively found in either males or females, in many instances male scat showed only very small proportions of Scorpaeniformes, Perciformes, Pleuronectiformes, and Gasterosteiformes whereas female diet proportions of Gadiformes and Salmoniformes, while smaller than in males, were still appreciable (Figure 3). This pattern resulted in uniformly lower Shannon diet diversity in males as compared to females (Figure 4). The best supported generalized linear models for variation in diet diversity all included sex with sex + season and sex + season + site performing marginally better than a model including only sex (Table 4). Male samples in particular, were less diverse in the late season compared to the early season, reflecting a greater proportion of adult salmon in the male diet (Figure 4). Comparisons of prey species richness yielded a similar result. Female prey species richness was greater than male prey species richness in seven of eight season/site/year comparisons. Only in the early season at Cowichan Bay in 2012 males consumed 13 prey species whereas females consumed 12. (Supporting Information Appendix S2 and S3).

**Table 3:** PERMANOVA results of the average proportions of the seven most common prey orders consumed by harbor seals relative to site (Comox and Cowichan), year (2012 and 2013), season (May–July and Aug–Nov), and sex (male and female). *p*‐Values <0.05 in bold

	Df	Sums of Sqs	Mean Sqs	*F*	*R* ^2^	*p* (> *F*)
Sex	1	0.22511	0.225107	23.5593	0.26806	**<0.001**
Season	1	0.19125	0.191247	20.0156	0.22774	**<0.001**
Site	1	0.08327	0.083274	8.7153	0.09916	**<0.001**
Year	1	0.05015	0.050152	5.2488	0.05972	**0.006**
Sex x Season	1	0.09117	0.091170	9.5417	0.10856	**<0.001**
Sex x Site	1	0.02537	0.025366	2.6548	0.03021	0.075
Sex x Year	1	0.01274	0.012738	1.3331	0.01517	>0.1
Season x Site	1	0.05588	0.055881	5.8484	0.06654	**0.004**
Season x Year	1	0.02978	0.029784	3.1171	0.03547	**0.047**
Site x Year	1	0.02728	0.027283	2.8554	0.03249	0.064
Residuals	5	0.04777	0.009555		0.05689	

**Table 4:** Generalized linear models explaining variation in Shannon index of prey order diversity in diet relative to sex (male and female), season (May–July and Aug–Nov), year (2012 and 2013), and site (Comox and Cowichan Bay)

Model	AIC	AICc	Δ AICc	*w*_*i*_	*R* ^2^
Sex + Season^a^	−18.6	−14.9	0.0	0.275	0.699
Sex + Season + Site	−19.7	−13.7	1.3	0.147	0.753
Sex	−15.6	−13.6	1.3	0.140	0.590
Sex + Season + (Sex*year)	−22.2	−12.9	2.1	0.098	0.814
Sex + Season + Year	−18.6	−12.6	2.3	0.086	0.735
Sex + Site	−15.8	−12.2	2.8	0.069	0.643
Sex + Season + (Sex*Season)	−17.8	−11.8	3.2	0.056	0.721
Sex + Year	−15.0	−11.4	3.5	0.047	0.625
Sex + Season + Site + Year	−20.2	−10.8	4.1	0.036	0.788
Sex + Season + (Sex*Location)	−19.7	−10.4	4.6	0.028	0.782
Sex + Site + Year	−15.5	−9.5	5.4	0.018	0.679
Season	−3.2	−1.2	13.7	<0.001	0.110
Site	−2.2	−0.2	14.7	<0.001	0.053
Year	−1.9	0.1	15.0	<0.001	0.036

a Coefficients (95% coefficient confidence intervals, *p*‐values) for each variable in the best supported model “Sex + Season”: intercept: 1.640 (1.541–1.739, *p* < 0.001), Sex (Male): −0.296 (−0.410 to −0.181, *p* < 0.001), Season (Late): 0.128 (0.013–0.242, *p* = 0.048).

### 3.3 Harbor seal sex‐specific predation on Pacific salmon

**Figure 5:**
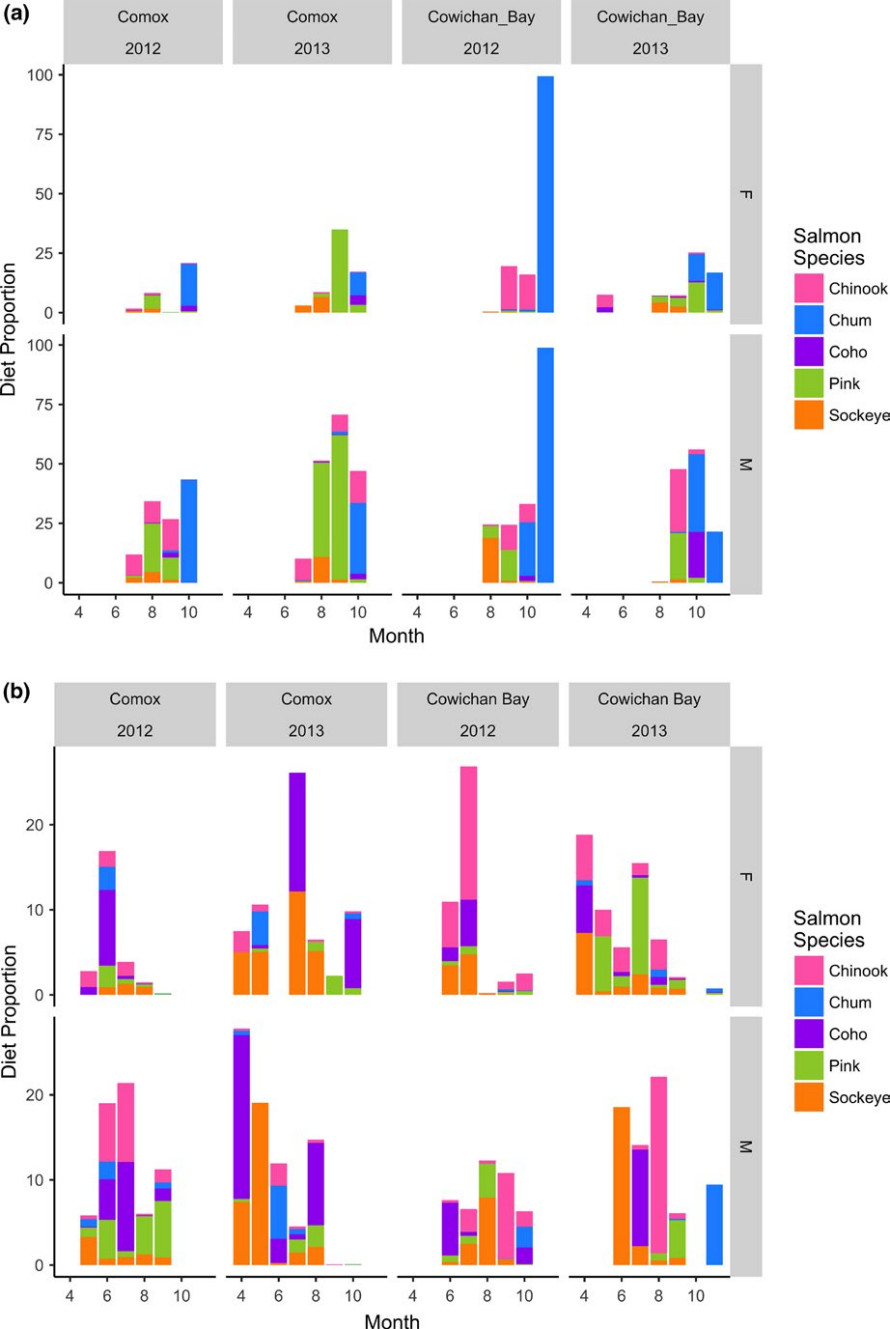
Mean monthly diet proportions of juvenile vs. adult Pacific salmon in female and male harbor seals. (a) Adult salmon prey. (b) Juvenile salmon prey. Average sequence proportions for all salmon species were added to obtain the displayed values. See Thomas et al. (2017) for details on the methodology for estimating juvenile vs. adult salmon diet proportions

**Figure 6:**
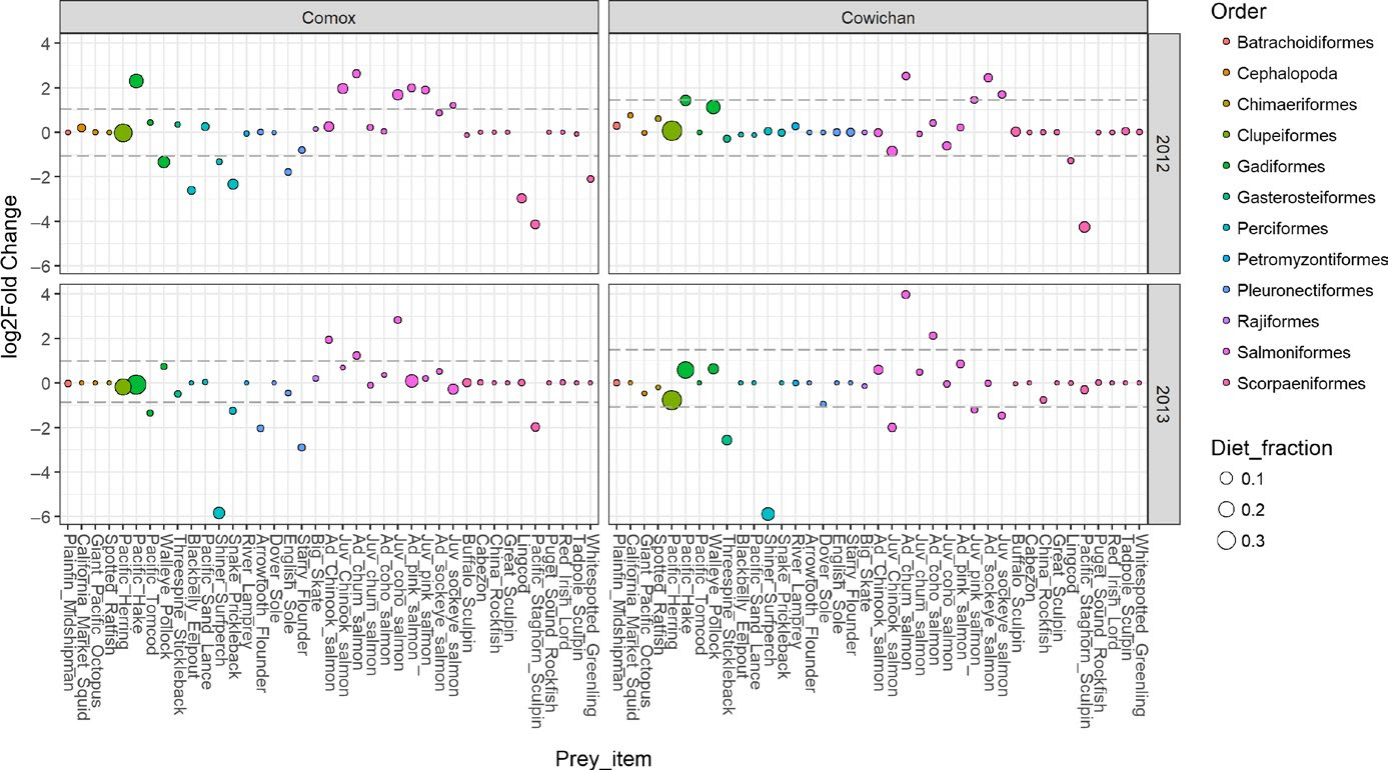
Relative sex‐specific differences of harbor seal diet proportion for prey species with a diet proportion >0.01 in at least one of the sexes in at least one year/site. Calculated log2 fold changes use females as the reference group. A positive LFC indicates a prey item eaten more by males, and a negative LFC indicates a prey item eaten more by females. Wald tests were used to determine if that LFC was significant (adjusted *p* < 0.01). Prey items above or below the dashed line were significantly different between the sexes. The size of the symbol indicates the mean proportion of the item in the combined diet of males and females

Male bias for salmon was most pronounced for adult salmon. During the late season and during August–October, the proportion of adult salmon in the male diet was consistently higher than in the female diet (Figure 5). This difference was due to male bias for all five Pacific salmon species (Figure 5). In November, only the haul‐out site at Cowichan Bay was sampled and the near identical proportions of adult salmon in the diet of both sexes in both years were dominated by adult Chum salmon (*O. keta*) (Figure 5). In October, proportion of Chum salmon in male diet was ca. three times higher than in female diet when adults from this prey species started appearing in substantial proportions in the diet. During 2013, both sexes showed higher diet proportions of adult pink salmon (*O. gorbuscha*) than in 2012, and this effect was most pronounced at Comox where adult pink salmon made up a larger proportion of the salmon prey. While male bias for adult pink salmon was statistically significant at Comox in 2012, the disproportionate increase of adult pink salmon in the female diet likely resulted in the absence of a significant bias in at the same site in 2013 (Figures 5 and 6). This large diet proportion of pink salmon in 2013 contributed to the significant interaction between season and year in the overall diet of seals (Table 3).

Sex‐specific bias for salmon was less consistent for juvenile salmon, but male bias still comprised seven of the ten significant sex‐specific differences in the proportion of juvenile salmon (Figure 6). Four of those significant male biases were observed at Comox in 2012: for juvenile Coho *(O. kisutch)*, pink, Chinook *(O. tshawytscha)*, and sockeye (*O. nerka*) salmon, when the mean monthly proportions for juvenile salmon in the male seal diet were consistently higher than the mean monthly proportions in the female diet (Figure 5). We further observed significant male biases for juvenile Coho at Comox in 2013 and for juvenile pink and sockeye at Cowichan Bay in 2012. In contrast, the only significant biases for juvenile salmon at Cowichan Bay in 2013 were in the female diet for juvenile Chinook, pink, and sockeye salmon (Figure 6).

### 3.4 Harbor seal sex‐specific predation by site and year

Although less important than sex and season, site and year were also significant factors in explaining diet variation at the order level (PERMANOVA: *R* ^2^ = 10%, *p* < 0.001 and *R* ^2^ = 6%, *p* = 0.008, respectively, Table 3). The higher diversity in diet orders for females at Comox (Figure 4) was due to the greater abundance of Scorpaeniformes, Perciformes, and Pleuronectiformes in the female diet with Pleuronectiformes almost being completely absent from the Cowichan Bay diet (Figure 3). In contrast, seals at Cowichan Bay showed a greater proportion of herring in their diet during the early season, which contributed to a significant interaction between site and season (Figure 3 and Table 3). The greater abundance of Scorpaeniformes, Perciformes, and Pleuronectiformes in the diet corresponded to more statistically significant instances of female‐biased predation on Pacific staghorn sculpin (*Leptocottus armatus*) and lingcod (*Ophiodon elongatus*) at both Comox and Cowichan Bay, and on snake prickleback (*Lumpenus sagitta*), blackbelly eelpout (*Lycodes pacificus*), arrowtooth flounder (*Atheresthes stomias*), English sole (*Parophrys vetulus*), starry flounder (*Platichthys stellatus*), and whitespotted greenling (*Hexagrammos stelleri*) at Comox only (Figure 6). The most extreme case of sex‐based predation in our study was the female preference for shiner surfperch (*Cymatogaster aggregata*) in 2013 (Figure 6). During that same year, we also detected female preference for three‐spined stickleback (*Gasterosteus aculeatus*) at Cowichan Bay, a species that was virtually absent in the diet of both male and female harbor seals in 2012 (Figure 3 and 6). A male preference for prey other than salmon was detected in 2012, when Pacific hake (*Merluccius productus*), which made up a substantial part of the harbor seal diet in both years and at both sites, occurred at much higher proportions in the male diet (Figure 6).

## 4 DISCUSSION

As expected, the seals in our study had a diverse diet indicative of a generalist predator. However, males and females represented two different generalists that showed consistent differences across two different sites and years, and that responded to seasonal changes in diet in a consistently distinct manner. Moreover, male diet was regularly less diverse than female diet and males specialized on a subsection of the female diet spectrum instead of feeding on a less diverse but separate set of prey species. In particular, males specialized on adult salmon when compared to females, lending our findings significance for understanding the impact of seal predation on endangered salmon runs in the Salish Sea.

### 4.1 Harbor seal sex‐specific diet

Results at the taxonomic order and species level of prey indicate differences in diet and foraging ecology between male and female harbor seals. Pacific herring was a favorite prey item of male and female harbor seals, a finding consistent with prior diet studies in the Salish Sea (Bromaghin et al., 2013; Lance et al., 2012). We were also able to parse out the differential impact of males and females on specific prey items to discern the foraging ecology of both sexes and expand findings collected with a smaller sample size (Bjorkland et al., 2015). Overall, males had higher diet proportions of pelagic species, particularly Pacific hake and adult salmon than females while the latter had higher proportions of benthic and estuarine species in their diet than the former. The diet proportions of juvenile salmon showed a more complex picture, with male diet proportions being significantly higher than female diet proportions in three of the site/year combinations except at Cowichan Bay in 2013. This last result may be the statistical effect of low male numbers at Cowichan Bay early in the season particularly during April (*n* = 1) and May (*n* = 3), months that were not been sampled in 2012. Visual inspection of proportions of juvenile salmon in the diet suggests that the impact of females on salmon was likely stronger on juveniles than adults, even though male bias still exists that may be quite substantial locally (Figure 5).

As central place foragers, the movements of harbor seals are related to the distance of the haul‐out site and the distribution of their prey species (Jones, Sparling, McConnell, Morris, & Smout, 2017). In the Salish Sea, the dive behavior of harbor seals indicates that males consistently undertake more shallow dives whereas females perform deeper dives, indicative of benthic foraging (Wilson, Lance, Jeffries, & Acevedo‐Gutiérrez, 2014). Such behavioral pattern is consistent with our findings that female diet contained a greater fraction of demersal or ground living fish in addition to pelagic fish, whereas males tended to specialize on pelagic prey.

Worldwide, harbor seals tend to move little, from dozens to 100 km from their haul‐out site (Blanchet, Lydersen, Ims, Lowther, & Kovacs, 2014; Suryan & Harvey, 1998; Thompson & Miller, 1990; Vincent et al., 2017), sometimes covering even longer distances (Björge, Oien, Hartvedt, Bothun, & Bekkby, 2002; Lesage, Hammill, & Kovacs, 2004; Lowry, Frost, Ver Hoef, & DeLong, 2001; Sharples, Moss, Patterson, & Hammond, 2012). Males appear to move farther and have larger core areas than females, at least in certain regions (Blanchet et al., 2014; Thompson et al., 1998). In the Salish Sea, males move further than females (Peterson, Lance, Jeffries, & Acevedo‐Gutiérrez, 2012) and genetic studies confirm this differential displacement (Burg, Trites, & Smith, 1999; Huber, Dickerson, Jeffries, & Lambourn, 2012); however, both males and females from estuarine sites tend to remain in a core area (Peterson et al., 2012). Coupled with our diet results, this information suggests that male seals consumed some of their prey in different areas than those in which females foraged.

Taken together, our results indicate that male and female harbor seals had different foraging ecologies. This finding is surprising given the relatively small differences in the movement and space use by the sexes (cited above), and slight sexual dimorphism of the species. Indeed, it has been suggested that the small sexual dimorphism may explain why sex was a poor predictor of both trip duration and distance in a large‐scale study of harbor seal movements around the British Isles (Sharples et al., 2012). On the other hand, male and females of seal species with a large sexual dimorphism (elephant seals, *Mirounga* spp. and gray seals, *Halicoherus grypus*) show noticeably differences in movement, space use, and foraging ecology (Breed et al., 2006; Le Boeuf et al., 2000).

Explanations for foraging differences by sex have been divided into five principal hypotheses, all of them linked to sexual dimorphism or differences in reproductive conditions (Wearmouth & Sims, 2008). Although our study was not designed to distinguish between these possible explanations, the social‐factors hypothesis, the predation‐risk hypothesis, and the thermal‐niche‐fecundity hypothesis appear less likely than the forage‐selection and activity‐budget hypotheses. Patterns in sex ratio at the two different sites were opposite, yet the overall patterns in sex‐specific diet differences remained the same arguing against the social‐factors hypothesis. The predation‐risk hypothesis indicates that the primary driver for female habitat choice would be the reduction of predation risk at the cost of suboptimal foraging conditions (Wearmouth & Sims, 2008). It is unlikely though that behavioral changes caused by mammal‐eating killer whales (*Orcinus orca*) (Deecke, Slater, & Ford, 2002) would result in consistent differences between males and females. Given the slight sexual dimorphism and endothermic metabolism of harbor seals, it seems unlikely that the thermal‐niche‐fecundity hypothesis explains our results.

It appears more likely that the forage‐selection and activity budget‐hypotheses apply to our study. Under the first hypothesis, the sexes use different prey items either because they are of different size or have different nutritional needs due to reproduction (e.g., nursing) and require different amounts of energy (Wearmouth & Sims, 2008). Under the second hypothesis, size may enable certain individuals to pursue prey (e.g., adult salmon) that smaller individuals cannot pursue, or care for offspring may keep females from investing the time to pursue large and mobile prey (Wearmouth & Sims, 2008). Compared with other seal species, harbor seal males are on average only moderately larger than females. In addition, the lack of sex‐specific differences in the use of adult chum salmon in November during both years at Cowichan Bay (there are no data available during this month at Comox) suggests that females are able to pursue adult salmon but limited to do so by caring for their offspring during other months. Thus, differences in reproductive biology between the sexes help explain our results. In contrast to males, females tend to isolate themselves to give birth, after which they must attend to their pups and select males with which to mate (Boness, Bowen, Buhleier, & Marshall, 2006; Coltman, Bowen, & Wright, 1999; Hayes et al., 2006). Pupping in region studied occurs from June to early August and is followed by ca. 1 month of nursing (Cottrell, Jeffries, Beck, & Ross, 2006). Weaned pups spend additional time (up to several months) in the area of their birth, but it is unclear whether this time is spent with or away from their mothers (Gaydos et al., 2013). These reproductive differences result in temporal variations in the energetic needs and spatial constraints of each sex (e.g., Boness et al., 2006).

The greater diet diversity in females compared to males may simply be the effect of females being more opportunistic foragers while being spatially constrained by their reproductive needs while males may be able to engage in more specialized foraging strategies that target large pelagic fish such as adult salmon and Pacific hake. In addition, females likely represent a mixture of reproductive and nonreproductive individuals (mainly juveniles), with the former pursuing local benthic prey while the latter may have a diet similar to males.

### 4.2 Harbor seal sex‐specific haul‐out use

In addition to sex‐specific differences in diet, our study also provided information about the sex ratio in haul‐out use. Such information is rarely obtained in harbor seals due to difficulties in sexing individuals via observation during counts from boats or the air. Consequently, studies on haul‐out use by sexes rely on tagged or photo‐identified individuals (e.g., Cordes & Thompson, 2013; Thompson, Fedak, McConnell, & Nicholas, 1989; Thompson, Miller, Copper, & Hammond, 1994).

We detected substantial changes in sex ratio during our annual sampling period that appeared to be the result of an actual turnover of individuals from both sexes instead of variation in the numbers of a “transient” sex being added to stable population of individuals from a “resident” sex. We do not know whether variation in total sample number reflected variation in haul‐out use by seals or was the result of variation in scat retention (e.g., caused by weather and wave action). Using haul‐out population sizes from the literature (Olesiuk, 2009), our monthly sample sizes at each site/year captured between 4 and 29% of individuals and need to be interpreted with some caution. Nevertheless, the change in sex ratio followed a repeatable pattern in both study years, with the pattern being reversed between the two study sites. These seasonal changes in the sex ratio between our two study sites may be explained by a combination of two factors: 1) the preference of male seals to eat adult salmon and juvenile salmon >10 cm in length and 2) the tendency of female seals to seek protected bays and inlets during the pupping season. The Comox haul‐out site is close in proximity to several rivers that produce large numbers of hatchery coho salmon, a species that rears in the river >1 yr prior to entering the salt water. An aggregative response by male seals to the pulsed hatchery releases of juvenile coho would explain the male bias in Comox during the spring months, and the relative lack of males in Cowichan Bay during the same period (Cowichan River Chinook are ocean‐type and emerge at small sizes <10 cm that are less preferred by seals (Thomas et al., 2017). Conversely, Cowichan Bay is spatially well‐protected from the open waters of the Strait of Georgia and may offer preferable pupping and rearing habitat for female harbor seals. This potential preference could explain why Cowichan Bay was biased toward female seals in the spring/early summer, compared with the more spatially exposed haul‐out site at Comox.

### 4.3 Strengths and limitations

We conducted a study of sex‐specific dietary differences of an unprecedented combination of both taxonomic, spatial, and temporal scale and resolution: 547 samples from two different sites during two years that were collected at monthly intervals between April and November and scored for prey diet proportions to the species level. We were also able to distinguish between stages (outmigrating juvenile vs. returning adults) for the salmon species in our sample. Most importantly, all samples were collected in a noninvasive manner, without the need to capture the animals.

Many previous studies have inferred diet differences based on differences in the spatial location and diving depth of the sexes (review by Wearmouth & Sims, 2008). Although we have detailed diet information at fine temporal scales, we do not know where harbor seals fed. The age class and body mass of the scat depositor are also unknown, both of which appear to be important factors influencing diet and foraging behavior (Bjorkland et al., 2015; Howard et al., 2013). One potential approach to address these limitations is to link movement studies of tagged individuals and scat analysis of their diet via genetic fingerprinting of samples to provide a full picture of diet and behavior (e.g., Jeanniard‐du‐Dot et al., 2017) and to refine bioenergetics models.

There is a potential systematic bias for underestimating the number of males inherent in the sex‐determining assay. We derived a crude estimate for the chance of misclassifying males as females of up to 4% (s. Materials and Methods). However, as the identified males were more “specialist” than females, this bias is not expected to have resulted in false sex‐specific differences. On the contrary, it is expected to have obscured such differences as males that were misidentified as females would make the estimated female diet more similar to the male diet. It is also possible that some individuals could be overrepresented in a sample. However, the relatively large sizes of the haul‐out sites (over 100 seals each) relative to the number of samples collected, decreases the probability of resampling individuals within each site (Rothstein, McLaughlin, Acevedo‐Gutiérrez, & Schwarz, 2017). The distance between both study sites (~140 km) is long enough, based on the fidelity to haul‐out sites (Hardee, 2008; Suryan & Harvey, 1998) and the movements of seals in the region (Peterson et al., 2012), to support the assumption that there was little movement of individuals, if any, between Comox and Cowichan Bay.

Relative correction factors (RCFs) have been developed when analyzing harbor seal scat samples to account for prey species‐specific biases (Thomas, Deagle, Eveson, Harsch, & Trites, 2016). However, given that we were interested in relative comparisons between males and females and characterized their diet from a numerical aggregate of many scat samples, prey species‐specific biases to DNA sequence counts are unlikely to fundamentally influence our results. Although it is conceivable that lower rates of DNA digestion for a prey item would amplify an already existing bias in diet proportions by a particular sex, we would not expect a qualitative change in the direction of such bias. For example, high lipid to protein ratios appear to inhibit DNA degradation and reads from lipid rich fish such as Pacific salmon are expected to be overrepresented compared to reads from low lipid fish like Pacific hake (Murray & Burt, 1983; Thomas, Jarman, Haman, Trites, & Deagle, 2014). Despite this difference both are overrepresented in the male diet even though their relative contributions may be skewed.

Finally, scat samples only represent a temporal snapshot of harbor seal predation as the passage rate of a diet item from stomach to scat is less than 2 days in harbor seals (Wilson, Greillier, & Hammond, 2017). While this may impact our estimate of the overall population diet it should affect the relative comparison of the sexes, especially as the overall trends persisted despite spatiotemporal variation in sex ratio.

### 4.4 Management implications

A common class of ecosystem models that are used to estimate the impact that predators have on prey populations are bioenergetic models (e.g., Chasco et al., 2017a,2017b). Such models typically ignore sex‐specific diet difference and assume a sex ratio of 1:1. The comparison of simple bioenergetics models using realistic model settings from the literature with models incorporating both sex‐specific diet proportions and sex ratio shows a difference of up to 8% when ignoring diet by sex (Supporting Information Appendix S4). In an actual data point from our study from April 2013 at Comox, we estimated that males made up 75% of samples and their diet consisted of 20% juvenile Coho salmon whereas females had no Coho in their diet. For this data point, a conventional model would have overestimated juvenile Coho consumption by 10%. It should be emphasized that small differences between models ignoring or incorporating diet by sex can yield large differences in the estimated number of individuals consumed depending on the prey species and life stage. For instance, a difference of 8% between both models miscounts by ca. 13,000 individuals the number of juvenile salmon consumed.

So far, our calculations have assumed that the conventional model uses local diet estimates. It is more common, however, to use global diet estimates that span wider geographic areas and time frames. For example, if we use the early season average diet proportions of juvenile Coho for males and females, ca. 6%, the conventional model underestimates the consumption of juvenile Coho in April by >50%. This effect is, however, mainly due to ignoring local spikes in prey proportions in the diet than to ignoring sex. Nevertheless, documenting focused predation for short time frames in specific locations by a specific sex, can be crucial to understanding the population dynamics of a prey species, and models that use wide‐ranging averages across time, space, and sex may be inadequate.

Our study adds to the evidence that harbor seals in the Salish Sea have some degree of foraging specialization that may occur over long time scales (Bjorkland et al., 2015; Bromaghin et al., 2013; Lance et al., 2012). They also suggest a complex food web between harbor seals and their prey and present a challenge for management (Bjorkland et al., 2015). Female harbor seals not only consume fewer salmonids than males but also they prey upon sculpins and other species that are major predators of salmon eggs and juveniles (Berejikian, 1995; Mace, 1983; Tabor, Chan, & Hager, 1998). It is then possible that female harbor seal consumption of sculpins and similar prey improves conditions for salmon, while male seals may have an opposite effect (Bjorkland et al., 2015). Given the magnitude of harbor seal predation on Chinook salmon populations relative to fisheries and other marine mammal predators (Chasco et al., 2017a,2017b), it is critical to understand the differential role that female and male harbor seals may have in the community, including indirect effects on endangered southern resident killer whales.

## 5 CONCLUSIONS

Our results show that our novel combination of techniques—noninvasive molecular bar coding of prey, age/stage determination via hard parts, and molecular sex identification from scat—allowed for both prey taxonomic and spatiotemporal resolution that is unprecedented in organisms notoriously difficult to study, like marine mammals. Specifically, we documented dietary differences in the diet of male and female harbor seals despite spatial and temporal variation, likely impacting prey species in distinct ways. Using sex‐specific diet data in food web models will incorporate the potential indirect effects of harbor seals on species of commercial interest, such as salmonids.

## AUTHOR CONTRIBUTIONS

D.S., A.T., and A.A‐G. conceived the study. A. T. collected the data. D. S., A. T., S. S., C. K., and T. K. analyzed the data and edited the manuscript. D. S., S. S., and A. A.‐G. wrote the manuscript.

## DATA ACCESSIBILITY

A database of prey diet proportions for each sexed scat sample is available at Dryad Digital Repository: https://doi.org/10.5061/dryad.g23j32s. For detailed information, on sampling locations and Genbank accession numbers for the diet bar coding sequences see Thomas et al. (2017).
