# Supplementary material for: Large‐scale molecular diet analysis in a generalist marine mammal reveals male preference for prey of conservation concern
Source: Ecol Evol. 2018 Sep 15;8(19):9889–905. doi: 10.1002/ece3.4474 (PMC6202700; doi:10.1002/ece3.4474)
Supplement: Supplementary file 1 [file ECE3-8-9889-s001.docx]

APPENDIX S1 Table 1. Monthly counts of male and female samples. E = early season, L = late season.

| Site | Year | Month | Season | N Males | N Females | N |
| --- | --- | --- | --- | --- | --- | --- |
| Comox | 2012 | 5 | E | 16 | 4 | 20 |
|  |  | 6 | E | 18 | 11 | 29 |
|  |  | 7 | E | 22 | 13 | 35 |
|  |  | 8 | L | 11 | 9 | 20 |
|  |  | 9 | L | 8 | 15 | 23 |
|  |  | 10 | L | 9 | 18 | 27 |
|  | 2013 | 4 | E | 13 | 4 | 17 |
|  |  | 5 | E | 5 | 12 | 17 |
|  |  | 6 | E | 8 | 5 | 13 |
|  |  | 7 | E | 11 | 7 | 18 |
|  |  | 8 | L | 10 | 10 | 20 |
|  |  | 9 | L | 9 | 16 | 25 |
|  |  | 10 | L | 7 | 11 | 18 |
| Cowichan Bay | 2012 | 6 | E | 8 | 11 | 19 |
|  |  | 7 | E | 19 | 15 | 34 |
|  |  | 8 | L | 13 | 7 | 20 |
|  |  | 9 | L | 7 | 19 | 26 |
|  |  | 10 | L | 16 | 13 | 29 |
|  |  | 11 | L | 4 | 2 | 6 |
|  | 2013 | 4 | E | 1 | 10 | 11 |
|  |  | 5 | E | 3 | 11 | 14 |
|  |  | 6 | E | 4 | 9 | 13 |
|  |  | 7 | E | 7 | 8 | 15 |
|  |  | 8 | L | 4 | 15 | 19 |
|  |  | 9 | L | 10 | 12 | 22 |
|  |  | 10 | L | 9 | 12 | 21 |
|  |  | 11 | L | 8 | 7 | 15 |

APPENDIX S2 Table 1. Prey species richness for by sex (male=M, female =F), season (April-July=E, August-November=L), site, and year. Only species with an average diet proportion of at least 0.01 in each of the combinations were counted. The sex with the higher prey species richness in each season/site/year combination is indicated by a bold letter.

| Sex | Season | Site | Year | Prey Species Richness |
| --- | --- | --- | --- | --- |
| **F** | L | Comox | 2012 | 16 |
| M | L | Comox | 2012 | 10 |
| **F** | E | Comox | 2012 | 19 |
| M | E | Comox | 2012 | 15 |
| **F** | L | Comox | 2013 | 16 |
| M | L | Comox | 2013 | 8 |
| **F** | E | Comox | 2013 | 14 |
| M | E | Comox | 2013 | 11 |
| **F** | L | Cowichan Bay | 2012 | 13 |
| M | L | Cowichan Bay | 2012 | 11 |
| F | E | Cowichan Bay | 2012 | 12 |
| **M** | E | Cowichan Bay | 2012 | 13 |
| **F** | L | Cowichan Bay | 2013 | 13 |
| M | L | Cowichan Bay | 2013 | 11 |
| **F** | E | Cowichan Bay | 2013 | 14 |
| M | E | Cowichan Bay | 2013 | 9 |

APPENDIX S2 Table 2. Comparison of generalized linear models of the prey species richness by sex, season, site, and year. The models are listed in descending order of support based on AICc values.

| Model | AIC | AICc | ΔAICc | w_i_ | R^2^ | |
| --- | --- | --- | --- | --- | --- | --- |
| Sex^a^ | 75.0 | 77.0 | 0.0 | 0.271 | 0.429 | |
| Sex+Site | 74.4 | 78.0 | 1.0 | 0.163 | 0.516 | |
| Sex+Year | 74.4 | 78.0 | 1.0 | 0.163 | 0.516 | |
| Sex+Site+(Sex*Site) | 73.2 | 79.2 | 2.2 | 0.088 | 0.602 | |
| Sex+Site+Year | 73.2 | 79.2 | 2.2 | 0.088 | 0.602 |  |
| Sex+Season | 75.8 | 79.4 | 2.4 | 0.080 | 0.471 | |
| Sex+Season+Site | 74.9 | 80.9 | 4.0 | 0.038 | 0.557 | |
| Sex+Season+Year | 74.9 | 80.9 | 4.0 | 0.038 | 0.557 | |
| Sex+Year+(Sex*Year) | 75.5 | 81.5 | 4.5 | 0.028 | 0.541 | |
| Sex+Season+Site+Year | 73.5 | 82.8 | 5.8 | 0.015 | 0.643 | |
| Sex+Season+(Sex*Season) | 77.0 | 83.0 | 6.0 | 0.013 | 0.496 | |
| Site | 82.5 | 84.5 | 7.5 | 0.006 | 0.086 | |
| Season | 83.3 | 85.3 | 8.3 | 0.004 | 0.041 | |
| Year | 82.5 | 85.3 | 8.3 | 0.004 | 0.086 | |

^a^ Coefficients (95% coefficient confidence intervals, p-values) for each variable in the best supported model “Sex”: intercept: 14.625 (13.077 – 16.173, p<0.001), Sex (Male): -3.625 (-5.814 - -1.436, p<0.006).

**APPENDIX S3**

APPENDIX S3 Figure S1. Effects of ignoring sex of in bioenergetic models. The basic principle of bioenergetics models is to use the energy requirements of a predator and to multiply them by the number of predators in the population and the relative proportion of the prey in the diet. The numerator then represents that proportion of the total energy requirement of the population that is being met by a particular prey species. Dividing this number by an estimate of the energy content per prey item provides an estimate of the number of prey individuals consumed. From this study, however, we know that males and females use prey differently and that sex ratio varies spatio-temporally; we also know that the sexes have different energy demands (Howard et al., 2013). Although unable to separate scat samples by males and females, traditional studies take into account sex-specific energy demands. This is because the total number of seals is not affected by ignoring sex and the diet fraction can be modeled as a weighted average of the unknown diet fractions from both male and female samples, which are then incorporated in the total diet fraction estimate for the population. However, because the sex ratio is typically unknown, models simply assume a 1:1 sex ratio and average the energy needs of the two sexes. Therefore, if the male and female energy needs differ and the numbers of male and female seals are not equal, ignoring sex will bias the model. This is also the case when energy needs differ and the diet fractions in the sexes differ, as the diet fractions are not multiplied by the correct energy needs. In the following, we examine the impact of ignoring both sex-specific diet proportions and sex ratio. Female diet proportion for prey item for which consumption is being estimated is set to 0.1. The deviation from the conventional bioenergetics model is shown as a function of the proportion of the prey item for which consumption is being estimated in the male diet. Em = male energy need. Ef = female energy need. Nm = number of males. Nf = number of females. Blue: energy need for both sexes = 16MJ/day, male proportion = 0.5 or 0.7. Red: female energy need = 16MJ/day, male energy need = 13.6MJ/day, male proportion = 0.5. Green: female energy need = 16MJ/day, male energy need = 13.6MJ/day, male proportion = 0.7. The blue line shows that when the energy needs of males and females don’t differ, differences in diet fraction result in no bias when sex is ignored, this holds true regardless of whether male and female numbers are the same or not. When we use actual estimates of female and male energy needs during the “early” season in the Salish Sea (Howard et al., 2013), a male bias for a particular prey item results in the conventional model overestimating the consumption of the particular prey species in question as we are disproportionally applying the higher energy estimate for females in the conventional model. This effect is increased by a higher proportion of males in the population.

Model details:

C = Number of prey items consumed by population (conventional model)

C_sex_ = Number of prey items consumed by population (sex-specific model)

E_f_ = Energy need of females

E_m_ = Energy need of males

N_f_ = Number of females

N_m_ = Number of males

D_f_ = Diet fraction in female diet

D_m_ = Diet fraction in male diet

P = Energy per prey item

Conventional bioenergetic model:

$$C=\frac{\left( \frac{E_{f}+E_{m}}{2} \right)\times{(N}_{m}+N_{f}) \times\left( \frac{D_{m}\times N_{m}+D_{f}\times N_{f}}{N_{m}+N_{f}} \right)}{P}$$

Sex-specific bioenergetics model:

$$C_{sex}=\frac{E_{f} \times N_{f} \times D_{f}}{P}+\frac{E_{m} \times N_{m} \times D_{m}}{P}$$
