# Supplementary figures and images for: Large‐scale molecular diet analysis in a generalist marine mammal reveals male preference for prey of conservation concern
Source: Ecol Evol. 2018 Sep 15;8(19):9889–905. doi: 10.1002/ece3.4474 (PMC6202700; doi:10.1002/ece3.4474)

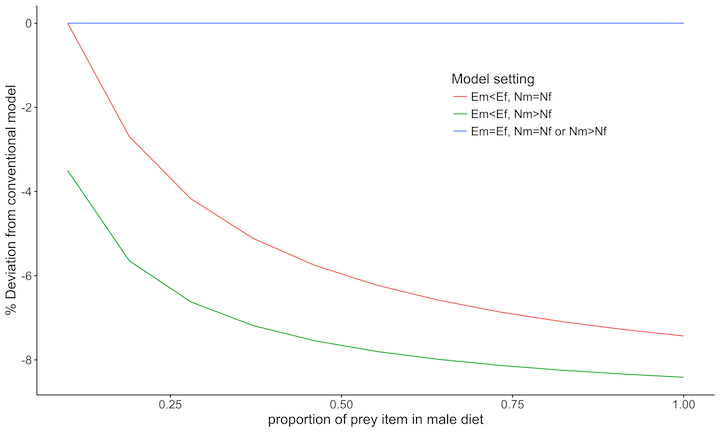

Supplement: Supplementary file 2 [file ECE3-8-9889-s002.tiff]
